# A novel conserved B-cell epitope in pB602L of African swine fever virus

**DOI:** 10.1007/s00253-023-12921-6

**Published:** 2024-01-09

**Authors:** Jinxing Song, Mengxiang Wang, Lei Zhou, Panpan Tian, Junru Sun, Zhuoya Sun, Chenyun Guo, Yanan Wu, Gaiping Zhang

**Affiliations:** 1 International Joint Research Center of National Animal Immunology, College of Veterinary Medicine, Henan Agricultural University, Zhengzhou, 450046 China; 2 Longhu Laboratory, Zhengzhou, 450046 China; 3 Agriculture Sciences, Peking University, Beijing, 100871 China

**Keywords:** African swine fever virus, pB602L, Monoclonal antibody, Epitope, Serological diagnosis

## Abstract

**Abstract:**

African swine fever virus (ASFV) is a complex DNA virus and the only member of the Asfarviridae family. It causes high mortality and severe economic losses in pigs. The ASFV pB602L protein plays a key role in virus assembly and functions as a molecular chaperone of the major capsid protein p72. In addition, pB602L is an important target for the development of diagnostic tools for African swine fever (ASF) because it is a highly immunogenic antigen against ASFV. In this study, we expressed and purified ASFV pB602L and validated its immunogenicity in serum from naturally infected pigs with ASFV. Furthermore, we successfully generated an IgG2a κ subclass monoclonal antibody (mAb 7E7) against pB602L using hybridoma technology. Using western blot and immunofluorescence assays, mAb 7E7 specifically recognized the ASFV Pig/HLJ/2018/strain and eukaryotic recombinant ASFV pB602L protein in vitro. The ^474^SKENLTPDE^482^ epitope in the ASFV pB602L C-terminus was identified as the minimal linear epitope for mAb 7E7 binding, with dozens of truncated pB602l fragments characterized by western blot assay. We also showed that this antigenic epitope sequence has a high conservation and antigenic index. Our study contributes to improved vaccine and antiviral development and provides new insights into the serologic diagnosis of ASF.

**Key points:**

• *We developed a monoclonal antibody against ASFV pB602L, which can specifically recognize the ASFV Pig/HLJ/2018/ strain.*

• *This study found one novel conserved B-cell epitope* ^*474*^*SKENLTPDE*^*482*^*.*

• *In the 3D structure,* ^*474*^*SKENLTPDE*^*482*^ *is exposed on the surface of ASFV pB602L, forming a curved linear structure.*

## Introduction

African swine fever (ASF) is one of the most complex infectious diseases in pigs, which was first identified in Kenya in 1921 and spreading in 2018, and it spread to the People’s Republic of China, a large pig-raising country, with severe economic losses (Ge et al. 2018). It was introduced to the Dominican Republic in July 2021, later to Haiti, and re-emerged in mainland Italy in January 2022. It is currently distributed in many sub-Saharan African countries, the Russian Federation, Transcaucasia, some Eastern and Central European countries, Sardinia, and Southeast and East Asia (Dixon et al. 2019a; Kolbasov et al. 2018; Netherton et al. 2019).

The causative agent is the African swine fever virus (ASFV), which is a large, enveloped double-stranded DNA virus that contains a genome of approximately 170–193 kbp, with inverted terminal repeats on both sides. The genome contains 150–167 genes, including genes involved in viral replication and assembly, regulation of host cell function, and immune escape. However, the functions of approximately 40 ASFV proteins remain unknown (Alejo et al. 2018; Dixon et al. 2019b). ASFV is the only known member of the Asfarviridae family and the *Asfivirus* genus (Alonso et al. 2018). The disease has a mortality rate of up to 100% and spreads rapidly due to its significant health and socioeconomic impact on international trade in pigs and pork products, and is a notifiable disease to the World Organization for Animal Health (WOAH) (Ward et al. 2021).

The emergence of attenuated strains of ASFV in many countries and their rapid spread pose serious challenges for ASFV prevention and control measures in domestic and wild pigs (Lv et al. 2021). However, the emergence of genotype I and recombinant strain variants has increased transmission and mortality rates, and unfortunately, a live attenuated vaccine derived from the ASFV genotype does not provide protection against recombinant viral attack (Zhao et al. 2023). Thus, the emergence of naturally occurring recombinant ASFV strains may pose new challenges to the global swine industry. Overall, the global spread of ASFV is highly complex and has serious implications for the global swine industry and related economies (Forth et al. 2023; Kitamura et al. 2023).

Vaccine development or treatment options for ASFV have been hampered by viral genome complexity, insufficient information on host-virus interactions, and the many unidentified genes in the genome (Muñoz-Pérez et al. 2021; Wu et al. 2021; Zheng et al. 2022). In the absence of a vaccine, early diagnosis is a critical prevention strategy.

pB602L is a late-stage non-structural protein that is not part of the viral particle, but is a highly immunogenic protein of ASFV that may be used to develop diagnostic tools (Epifano et al. 2006). As a molecular chaperone, it participates in the folding of the p72 nucleocapsid protein and is located outside the virus factory. The formation of the p72 trimeric structure is dependent on the co-expression of pB602L (Cobbold et al. 2001; Liu et al. 2019). Importantly, the absence of pB602L from the genome results in an abnormal zipper-like structure that is unable to form icosahedral virus particles (Epifano et al. 2006). Previous studies have reported that pB602L elicits strong and specific responses to the serum from convalescent ASFV (Gallardo et al. 2009; Gutiérrez-Castañeda et al. 2008). In addition, pB602L can be used to distinguish pigs which are naturally infected with wild ASFV strains from pigs immunized with structural protein subunit vaccines (Gutiérrez-Castañeda et al. 2008; Irusta et al. 1996). Immunoglobulin type G (IgG) monoclonal antibodies (mAbs) are important reagents used for diagnostic and therapeutic purposes. However, the molecular basis of pB602L antigenicity and the mechanism behind its effects on p72 remains unclear. To date, very few studies have thus far generated specific mAbs against the pB602L protein and mapped its epitopes, limiting basic research on pB602L.

To better understand the biological functions of ASFV pB602L, we used purified recombinant HIS-tagged pB602L protein and tested its ability to induce B-cell immunogenicity in pig serum after natural infection with ASFV. Using hybridoma technology, we generated mAb 7E7 that specifically bound to the ASFV Pig/HLJ/2018 strain. Due to the high specificity between both reagents, we identified a B-cell linear epitope (^474^SKENLTPDE^482^) recognized by mAb 7E7. Additionally, homology analyses showed that B-cell epitope sequences were highly conserved in several ASFV strains, including I, II, I and II recombinant, VIII, X, and XI genotype isolates. Our molecular docking approach showed that contact residues between ^474^SKENLTPDE^482^ and mAb 7E7 formed various interactions, including salt bridges, hydrogen bonds, and hydrophobic interactions, which enhanced the affinity of the antibody to an antigen. Thus, we provided fundamental insights and practical applications to ASFV.

## Materials and methods

### Serum samples

Healthy swine-negative serum and standard positive sera for ASFV were obtained from the China Veterinary Culture Collection Center (Beijing, China).

### ASFV pB602L recombinant cloning, expression, and purification and detection of pB602L-specific antibodies in porcine serum samples

Specific primers 5′-TGGTGGTGCTCGAGTGCGGCCGCCAATTCTGCTTT-3′ (F) and 5′-ATGGGTCGCGGATCCGAATTCATGGCAGAATTTAA-3′ (R) were designed based on the published ASFV *B602L* gene sequence of ASFV (GenBank MK333180.1). Primers and genes were synthesized by Tsingke Biotechnology Co., Ltd. (Beijing, China). The amplified ASFV *B602L* DNA was subcloned into the expression vector pET-28a (+) (Novagen, San Diego, CA, USA) using *Not*I and *Eco*RI restriction enzyme sites to yield the recombinant plasmid pET-28a (+)-*B602L*. Then, the plasmid was chemically transformed into *Escherichia coli* BL21 (DE3) TSsetta cells (Tsingke, Beijing, China). Once the OD_600_ reached 0.4–0.6, cells were induced with 0.5 mM isopropyl β-D-1-thiogalactopyranoside (IPTG) in Luria-Bertani medium at 16 °C overnight. To isolate a HIS-tagged ASFV pB602L recombinant protein, cell lysates were purified using Ni-Sepharose 6 Fast Flow resin (GE Healthcare, Chicago, IL, USA), proteins were eluted in different imidazole concentrations, and purified proteins were analyzed by western blot (WB) and sodium dodecyl sulfate-polyacrylamide gel electrophoresis (SDS-PAGE). pB602L-specific antibody titers from serum samples were determined by enzyme-linked immunosorbent assay (ELISA) and pB602L reactivity was verified by WB using ASFV-positive serum (1:1000 dilution).

### IgG mAb generation and identification

We intraperitoneally inoculated 8-week-old female BALB/c mice (Changsheng, Liaoning, China) with purified recombinant pB602L protein (100 μg/mouse) mixed with QuickAntibody™-Mouse 5W rapid immune adjuvant (BioDragon, Beijing, China) on days 0 and 21. At week 4, tail vein blood was collected and antibody titers were determined by ELISA. Mice with the highest responses by ELISA were selected for hybridoma generation, and 3 days later, total spleen cells were harvested and processed by polyethylene glycol-mediated fusion to generate hybridomas.

We generated mAbs according to standard procedures (Galfrè et al. 1981). First, fusion cells were cultured in HAT medium (Sigma, St. Louis, USA) for 6 days, and fused cells were selected in complete HAT medium. Several rounds of limiting dilution cloning were performed and hybridoma cells were tested by ELISA for antibody production against recombinant pB602L. Briefly, purified recombinant HIS-tagged ASFV pB602L protein was diluted to 4 μg/mL. ELISA plates were coated with bicarbonate buffer overnight at 4 °C. After three washes in phosphate-buffered saline (PBS) plus 0.01% Tween-20 (PBST), plates were blocked in PBS plus 5% skim milk for 1 h at room temperature (RT). Plates were then washed three times, and then, 100-μL hybridoma supernatant was added and incubated at 37 °C for 1 h. SP2/0 cell supernatants and mouse-positive serum were used as negative and positive controls, respectively. After washing three times in PBST, horseradish peroxidase (HRP) coupled to goat anti-mouse IgG secondary antibody (Proteintech, Wuhan, China, 1:5000 diluted in 5% milk/PBST) was added and incubated for 1 h at 37 °C. After washing in PBST, HRP was detected with 3,3′,5,5′-tetramethylbenzidine (Solarbio, Beijing, China), and reactions were stopped by the addition of 50 μL sulfuric acid (2 M). The absorbance at 450 nm was then read on a microplate reader (TECAN, Männedorf, Switzerland). P/N ≥ 2.1 (positive/negative value, P/N value) was used as a positive criterion and was used to screen and select individual hybridoma clones that stably secrete mAbs against the ASFV pB602L protein. Monoclonal cells were cultured in Dulbecco’s modified Eagle medium (DMEM) supplemented with 1% penicillin-streptomycin (Solarbio, Beijing, China) and 10% FBS (Thermo Fisher Scientific, Waltham, Massachusetts). For further mAb production, ascites was collected from female BALB/c mice females (8 weeks old). SDS-PAGE was used to separate antibody light and heavy chains and Coomassie Blue was used for staining. mAbs were purified by protein A affinity chromatography and antibody titers in ascites were determined by indirect ELISA. Analysis of mAb 7E7 subtype was performed using a mouse mAb isotyping test kit (Protech, Wuhan, China) according to the manufacturer’s protocols.

### Functional mAb verification

Inactivated porcine alveolar macrophages (PAMs) infected with the ASFV Pig/HLJ/2018 were provided by the Harbin Veterinary Research Institute (China) and stored at −80 °C. We used WB to assess whether the mAbs specifically bound to the ASFV Pig/HLJ/2018 strain. Briefly, inactivated samples were separated by SDS-PAGE and transferred to polyvinylidene fluoride membranes (Millipore, Darmstadt, Germany). The membrane was blocked with 5% nonfat milk in TBS buffer containing 0.1% (w/v) TBST for 1 h at RT. The membranes were then incubated overnight at 4 °C with hybridoma supernatant (primary antibody). The next day, the membranes were washed three times, and the goat anti-mouse IgG-HRP antibody (Proteintech, Wuhan, China) was added as a secondary antibody for 1 h at RT. Blots were incubated with enhanced chemiluminescence (ECL) solution (New Cell & Molecular Biotech, Suzhou, China) for 1 min, and images were captured using a luminescence image analyzer (Amersham Imager 680, GE Healthcare Japan, Tokyo, Japan). mAb specificity was characterized by immunofluorescence assay (IFA) using 293T cells transfected with the pCMV14-3×Flag-*B602L* gene plasmids. After transfection for 24 h, 293T cells were fixed in 4% (v/v) formaldehyde for 15 min and permeabilized in 0.1% Triton X-100 (v/v) in 1×PBS for 15 min. Cells were osmotically fixed at RT for 10 min and blocked in 5% bovine serum albumin (Solarbio, Beijing, China) in 0.1% Triton/PBS for 1 h. The cells were then incubated with hybridoma supernatant (primary antibody) for 2 h, washed in PBS, and incubated for 1 h with secondary antibody anti-mouse fluorescein isothiocyanate dye (Proteintech, Wuhan, China, 1:500) in blocking buffer in the dark, and then washed three times in PBS and incubated with 4′,6-diamidino-2-phenylindole (Solarbio, Beijing, China) for 5 min; fluorescence images were captured by microscopy (Olympus, IX73, Tokyo, Japan).

### Epitope ASFV pB602L mapping

**Fig. 1:**
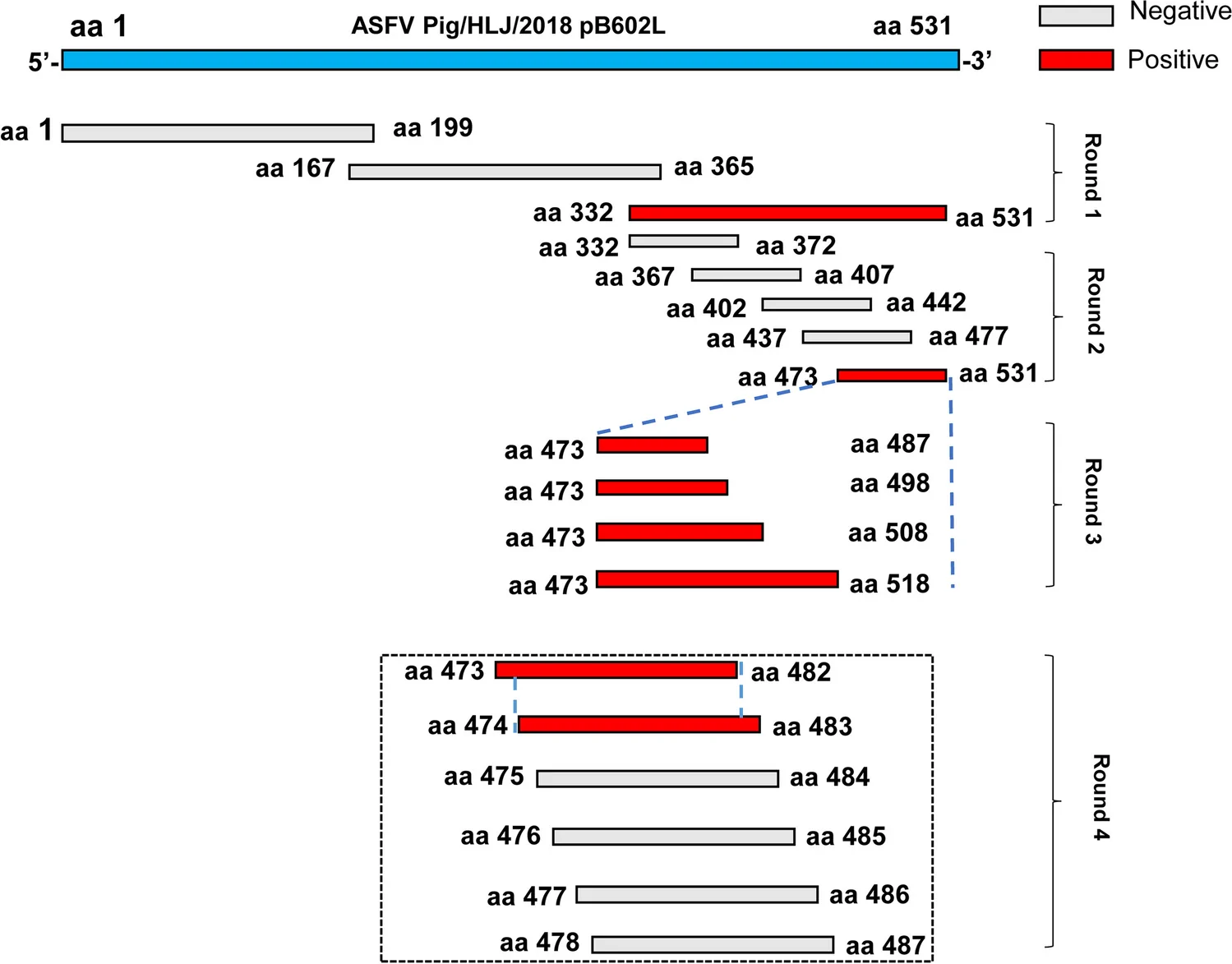
Scheme showing ASFV pB602L fragments used for B-cell epitope mapping. Identification of the mAb 7E7 binding epitope using four rounds of overlapping PCR. The original entire pB602L sequence (531 amino acids (aas)) is blue. Numbers in the figure represent aa positions in the pB602L protein and truncated proteins

We expressed several overlapping truncated pB602L fragments to identify ASFV pB602L epitopes. To construct the necessary serial vectors, full-length coding sequences and their various truncations of ASFV *B602L* were amplified and cloned into the vector pGEX-6p-1 (Amersham Biosciences, Piscataway, New Jersey) and expressed in *E. coli* as GST fusion proteins, after four rounds of truncation until the smallest binding domain was recognized by mAb 7E7 (Fig. 1). Primers are listed in Table 1. The recombinant proteins were identified by WB using a peroxidase-conjugated anti-GST antibody (Proteintech, Wuhan, China, 1:5000) and the resulting mAb 7E7.

**Table 1:** Study primers for plasmid construction

Primer	Sequence (5′-3′)	Amplicon size (bp)	Amino acid length range (aas)
1-1F	GGCCCCTGGGATCCCCGGAATTCATGGCAGAATTTAAT	1–597	1–199
1-1R	AGTCACGATGCGGCCGCTCGAGGCTTGTACAAGTGCTT		
1-2F	GGGCCCCTGGGATCCCCGGAATTCGACACTTGTGCAAG	499–1095	167–365
1-2R	TCACGATGCGGCCGCTCGAGCTTAAAGCTTCCCAGGAG		
1-3F	GGGGCCCCTGGGATCCCCGGAATTCCAAAAGGTTGATG	997–1593	333–531
1-3R	GTCAGTCACGATGCGGCCGCTTACAATTCTGCTTTTGT		
2-1F	GGGGCCCCTGGGATCCCCGGAATTCCAAAAGGTTGATG	997–1116	333–372
2-1R	TCAGTCACGATGCGGCCGCATTGTAGCGCTCTCTGTT		
2-2F	CTGGGATCCCCGGAATTCAGAGAGCGCTACAATTAT	1102–1221	368–407
2-2R:	TCAGTCACGATGCGGCCGCTTTTTTGTGGTAAATAGT		
2-3F	CCTGGGATCCCCGGAATTCATTTACCACAAAAAAGCA	1207–1326	403–442
2-3R	CAGTCACGATGCGGCCGCCTCCTTAAAGGAAAGCAG		
2-4F	CTGGGATCCCCGGAATTCCTTTCCTTTAAGGAGCTA	1312–1431	438–477
2-4R	CAGTCACGATGCGGCCGCATTTTCTTTACTAAGCAG		
2-5F	CTGGGATCCCCGGAATTCCTTAGTAAAGAAAATTTA	1417–1593	473–531
2-5R	GTCAGTCACGATGCGGCCGCTTACAATTCTGCTTTTGT		
3-1F	CTGGGATCCCCGGAATTCCTTAGTAAAGAAAATTTA	1417–1461	473–487
3-1R	CAGTCAGTCACGATGCGGCCGCTTATATCAGCTCGCT		
3-2F	CTGGGATCCCCGGAATTCCTTAGTAAAGAAAATTTA	1417–1494	473–498
3-2R	GTCAGTCACGATGCGGCCGCTTAAAGCGCATTATCTAA		
3-3F	CTGGGATCCCCGGAATTCCTTAGTAAAGAAAATTTA	1417–1524	473–508
3-3R:	CAGTCAGTCACGATGCGGCCGCTTATATCGTATCATC		
3-4F:	CTGGGATCCCCGGAATTCCTTAGTAAAGAAAATTTA	1417–1554	473–518
3-4R	AGTCAGTCACGATGCGGCCGCTTAATTATTATTGTT		
4-1F	CTGGGATCCCCGGAATTCCTTAGTAAAGAAAATTTA	1417–1446	473–482
4-1R	AGTCAGTCACGATGCGGCCGCTTCATCGGGGGTTA		
4-2F	GGCCCCTGGGATCCCCGGAATTCAGTAAAGAAAATTTA	1420–1449	474–483
4-2R	GTCAGTCACGATGCGGCCGCTTCTTCATCGGGGGT		
4-3F	CCCTGGGATCCCCGGAATTCAAAGAAAATTTAACC	1423–1452	475–484
4-3F	TCAGTCACGATGCGGCCGCGCTTTCTTCATCGGG		
4-4F	CCCTGGGATCCCCGGAATTCGAAAATTTAACCCCC	1426–1455	476–485
4-4F	GTCAGTCACGATGCGGCCGCCTCGCTTTCTTCATC		
4-5F	CCCTGGGATCCCCGGAATTCAATTTAACCCCCGAT	1429–1458	477–486
4-5R	GTCAGTCACGATGCGGCCGCCAGCTCGCTTTCTTC		
4-6F	CCCCTGGGATCCCCGGAATTCTTAACCCCCGATGAA	1432–1461	478–487
4-6R	CAGTCAGTCACGATGCGGCCGCTTATATCAGCTCGCT		

### Epitope conservation analysis

To determine the degree of epitope conservation across different ASFV genotypes for the pB602L protein, we compared the ^474^SKENLTPDE^482^ epitope with 17 reference ASFV strain sequences using Geneious software (version 2022.2.2, Auckland, New Zealand). All reference strain sequences were obtained from GenBank (https://www.ncbi.nlm.nih.gov/gene/).

### Structure modeling and molecular docking

In structural biology, it is important to predict specific protein structural features, such as three-dimensional (3D) structural features, that provide insight into biological function, e.g., to guide protein-protein interaction or epitope identification studies. First, secondary structures (β-strands, α-helix, and loops), surface probability, epitope residue hydrophilicity, and the antigenic index of the putative ASFV pB602L protein model were analyzed using PROTEAN software (DNAStar, Madison, Wisconsin). Molecular docking was performed to gain further insight into the interaction forces between antigen and antibody residues and identify key binding sites. The genes encoding for the variable regions of the heavy and light chains of mAb 7E7 were sequenced by Detai Biologics (Detai Biologics, Nanjing, China) and structure modeling designed from scratch using phyre2 (www.sbg.bio.ic.ac.uk/~phyre2 ). The structure was prepared using the structure preparation tool in Molecular Operating Environment (MOE, 2019.01; Chemical Computing Group, Montreal, Canada). PDB files of ASFV pB602L and mAb 7E7 PDB files were generated and optimized using the structure preparation module. Using interaction energies and geometric matching qualities as criteria for complex selection, the protein-protein docking module was used to comprehensively analyze interactions between pB602L and mAb 7E7. Structure visualization, molecular editing, and figure generation were performed in PyMOL 2.1 software (www.pymol.org ).

## Results

### Recombinant pB602L protein cloning, expression, and characterization

**Fig. 2:**
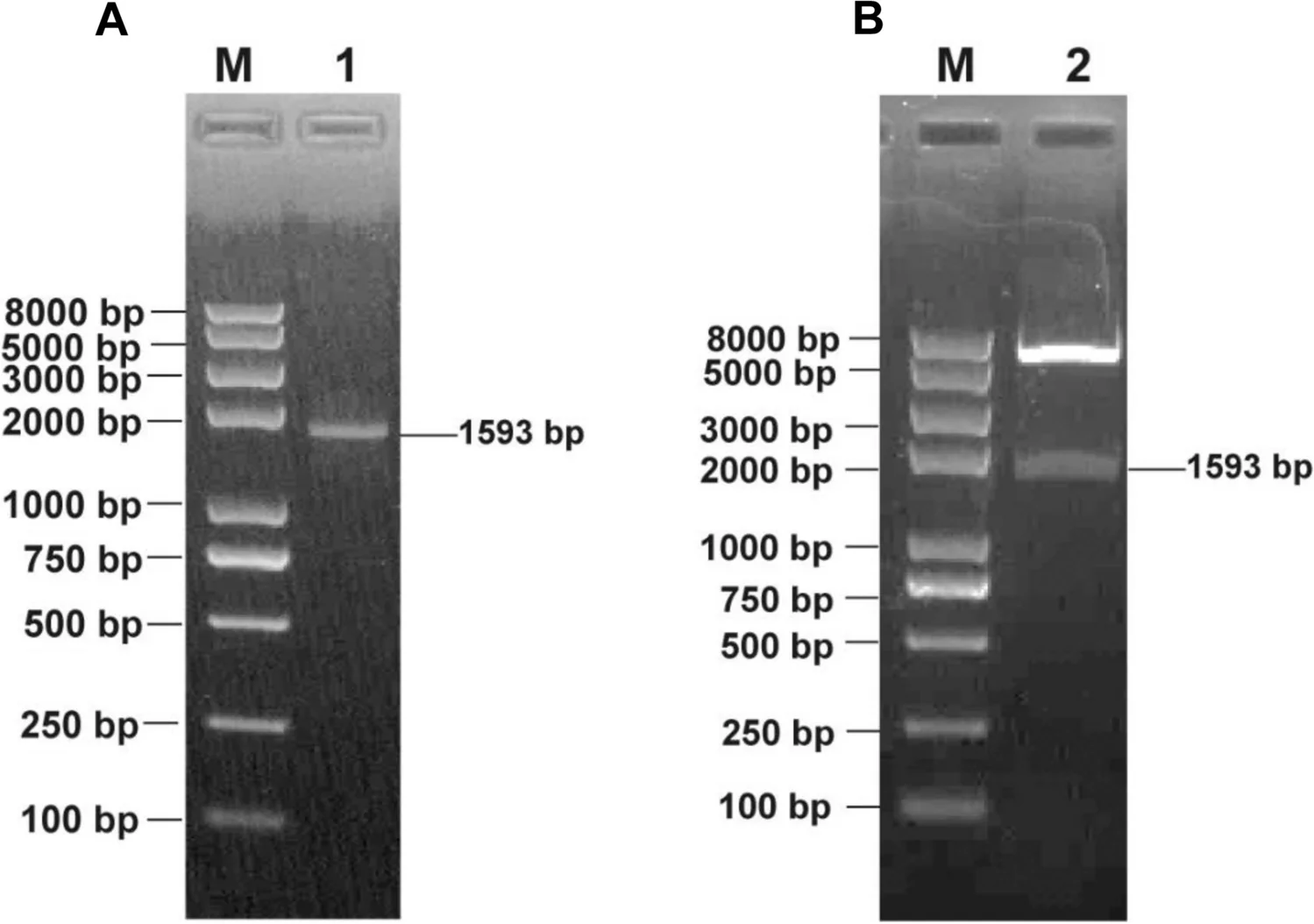
Identification of amplified products and recombinant plasmids expressing ASFV *B602L*. **A** Lane 1, amplified ASFV *B602L* products; fragment size = 1593 bp. **B** Lane 2, pET28a (+) products after digestion with *Not*I and *Eco*RI. Lane M, DNA marker

**Fig. 3:**
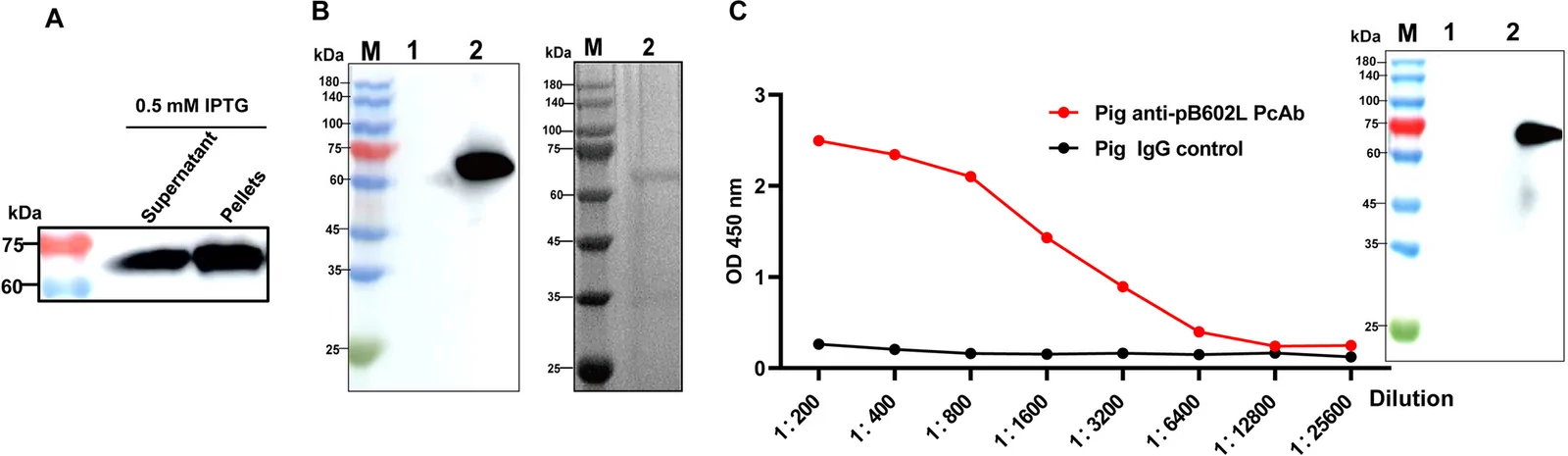
Expressed and purified ASFV pB602L protein and recombinant pB602L immunogenicity analyses. **A**, **B** WB and SDS-PAGE were used to analyze recombinant HIS-tag pB602L expression and purification. **C** Target proteins were examined using indirect ELISA and WB. Target protein immunogenicity was examined using ASFV-positive pig serum (ASFV Pig/HLJ/2018 infected pigs) and ASFV-negative pig serum (control group). M = protein marker; lane 1, empty pET28a plasmid; and lane 2, purified pB602L protein

The *B602L* gene sequence of ASFV was synthesized and amplified based on the reference strain ASFV Pig/HLJ/2018. As shown in Fig. 2A, a full-length *B602L* gene fragment (1593 bp) was amplified. After *Not*I*/Eco*RI double digestion, the restricted PCR products were separated by gel electrophoresis. The digested products are 1593 bp (Fig. 2B). The plasmid was transformed into *E. coli* BL21 (DE3) cells for recombinant protein production. Expressed as a soluble protein in the supernatant, recombinant ASFV pB602L was purified by nickel column chromatography and identified by SDS-PAGE, with an expected molecular weight of approximately 65 kDa (Fig. 3A). Protein reactivity was verified by anti-HIS mAb-mediated WB (Fig. 3B). pB602L immunogenicity was determined by testing antibody responses to pB602L in swine sera infected with the ASFV Pig/HLJ/2018 strain by indirect ELISA (Fig. 3C) and WB (Fig. 3D), which showed that the protein reacted well with ASFV-infected swine serum.

### WB and IFA analyses

**Fig. 4:**
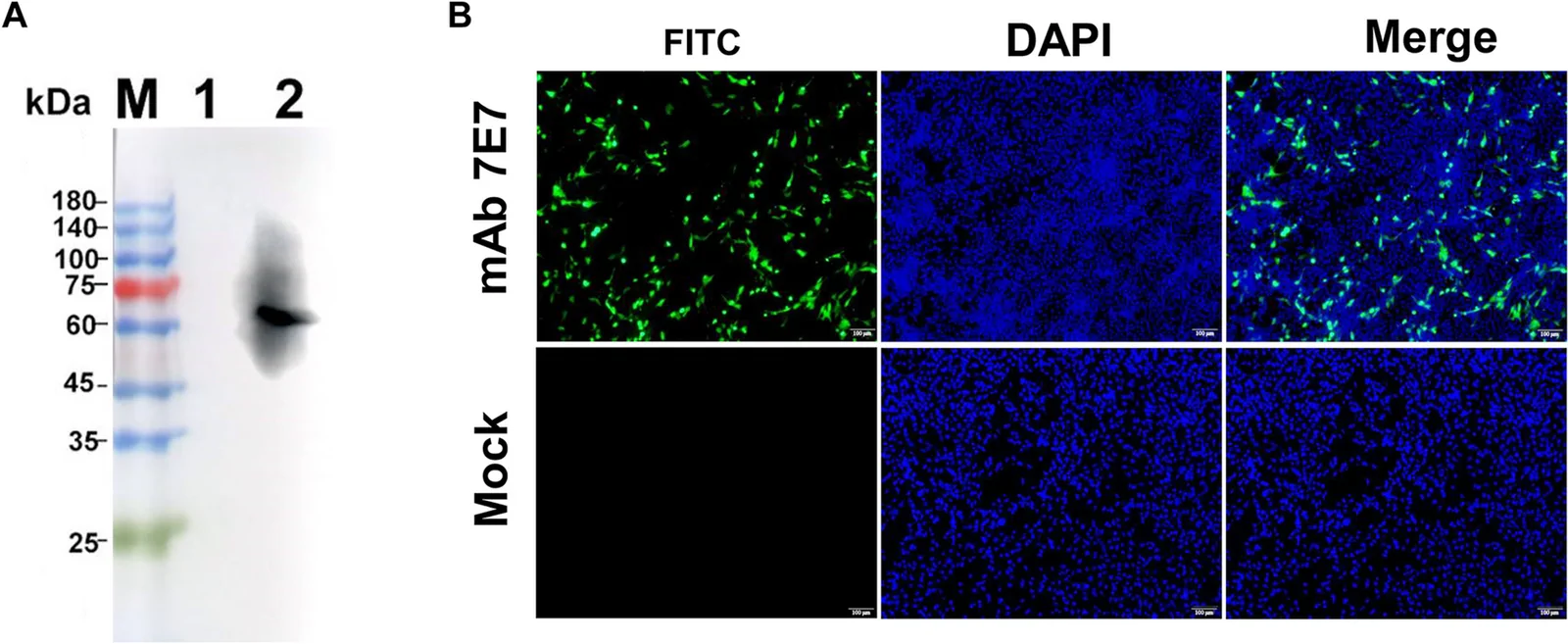
mAb 7E7 specificity analyses using WB and IFA. **A** pB602L mAb 7E7 specifically bound to the ASFV Pig/HLJ/2018 strain; lane M, protein marker; lane 1, uninfected PAM cells were used for empty control; and lane 2, samples of inactivated porcine alveolar macrophages infected with the ASFV Pig/HLJ/2018 strain. **B** 293T cells were transfected with plasmids encoding ASFV pB602L to confirm the reactivity of the mAb 7E7 against ASFV pB602L by IFA. Cell nuclei were visualized using 4′,6-diamidino-2-phenylindole under fluorescence microscopy

The specificity of the mAb against ASFV virions and pB602L protein was determined by WB and IFA. As shown in Fig. 4A, the results indicated that the mAb 7E7 reacted specifically with inactivated ASFV Pig/HLJ/2018 strain-infected PAMs. To further analyze the reactivity of mAb 7E7 against ASFV pB602L, IFA was performed to examine ASFV pB602L protein expression using mAb 7E7. IFA images show that no pB602L protein was detected in empty vector-transfected HEK-293T cells (negative control), whereas a clear and predominantly cytoplasmic staining pattern was observed in *B602L*-transfected cells. Thus, mAb 7E7 specifically bound to the ASFV Pig/HLJ/2018 strain and to the ASFV pB602L recombinant protein.

### mAb production and characterization against B602L

**Fig. 5:**
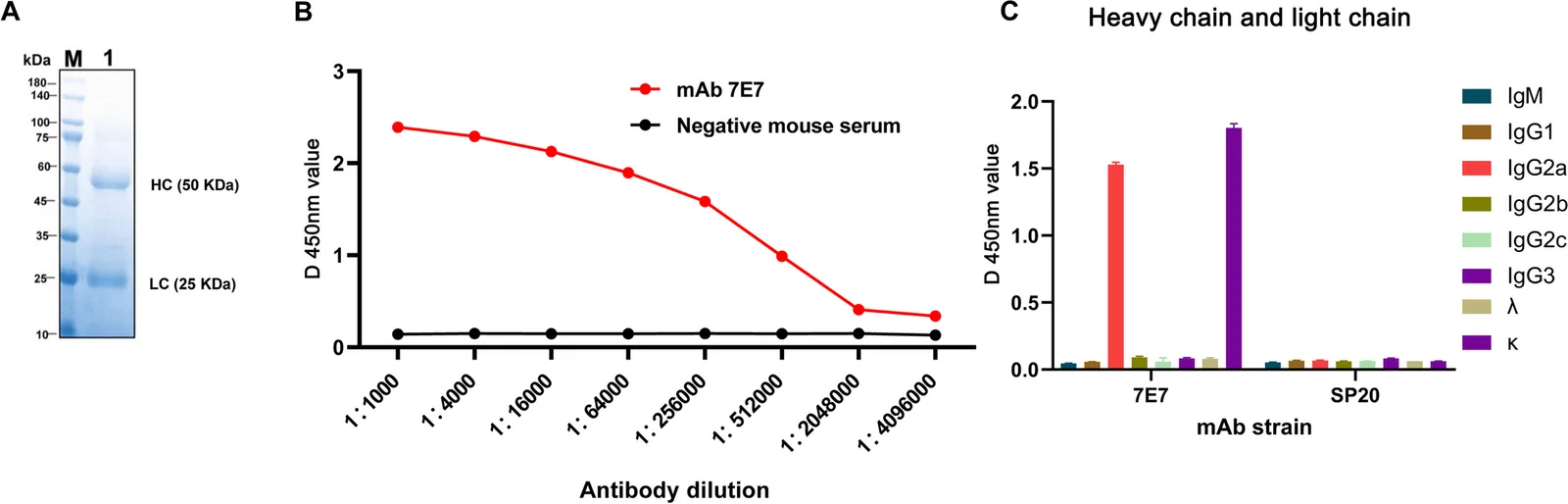
The identification of the purified mAb 7E7 by SDS-PAGE and the isotype by ELISA. **A** Lane M, protein marker; lane 1, purified ascites. HC and LC are immunoglobulin heavy and light chains, respectively. **B** Purified mAb 7E7 titration data. **C** Isotyping of the mAb 7E7, SP2/0 cell supernatants were used as negative controls

Using cell fusion techniques combined with restriction dilution, a hybridoma clone was generated that produced antibodies specific to pB602L. This stable, positive hybridoma clone (7E7) was selected to produce ascites, which were collected after 10–14 days and analyzed by SDS-PAGE. As shown in Fig. 5A, IgG heavy (HC) and light (LC) chains are indicated by 50 kDa and 25 kDa bands, respectively. pB602L levels in ascites were determined by indirect ELISA and showed high titers; end-point titers ranged from 1:1000 to 1:4,096,000 (Fig. 5B)*.* The heavy chain subclass of mAb 7E7 was identified as IgG2a and the light chain was identified as κ (Fig. 5C).

### Identify the precise epitope recognized by the mAb 7E7

**Fig. 6:**
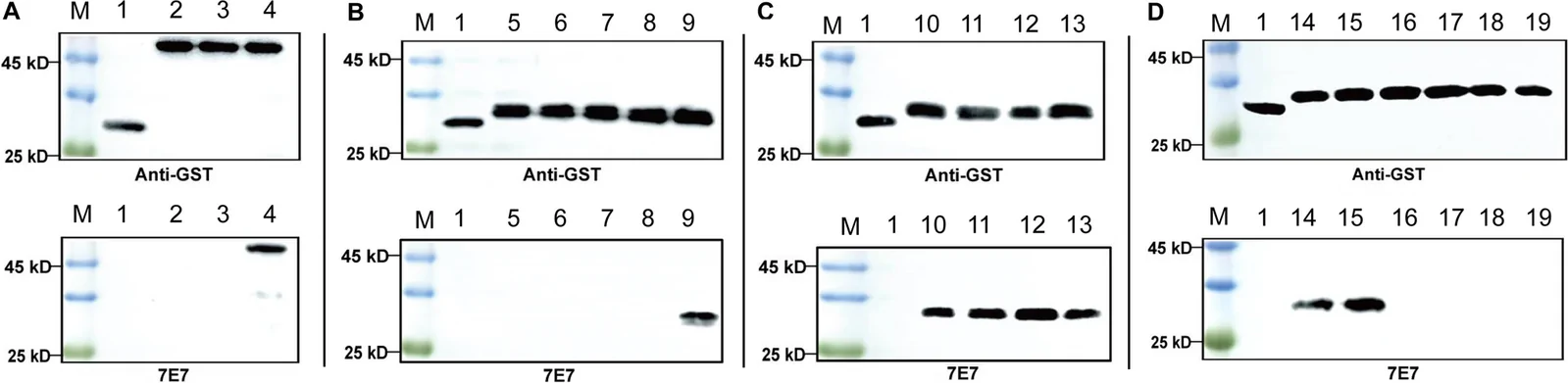
Precise mAb 7E7 epitope mapping. Several truncated pB602L fragments were cloned into pGEX-6p-1 and expressed as GST fusion proteins. GST fusion proteins were probed with anti-GST antibody by WB, and the results were also used as positive controls. **A** Lane 2, peptide 1–199 amino acids (aas) (pB602L); lane 3, peptide 167–365 aa; lane 4, peptide 332–531 aa. **B** Lane 5, peptide 332–372 aa; lane 6, peptide 367–407 aa; lane 7, peptide 402–442 aa; lane 8, peptide 437–477 aa; lane 9, peptide 473–531 aa. **C** Lane 10, peptide 473–487 aa; lane 11, peptide 473–498 aa; lane 12, peptide 473–508 aa; lane 13, peptide 473–518 aa. **D** Lane 14, peptide 473–482 aa; lane 15, peptide 474–483 aa; lane 16, peptide 475–484 aa; lane 17, peptide 476–485 aa; lane 18, peptide 477–486 aa; and lane 19, peptide 478–487 aa. Lane M, protein marker; lane 1, GST alone was used as a negative control

To map antigenic epitopes in the mAb 7E7, four rounds of overlapping short peptides spanning full-length pB602L were designed. The complete pB602L protein of ASFV was truncated into three overlapping fragments, aa 1–199, aa 167–365, and aa 332–531, which were solubly expressed as GST fusion proteins in *E. coli* BL21 (DE3) cells and detected by WB. The results showed that only aa 332–531 could react with mAb 7E7 (Fig. 6A), suggesting that the epitope binding for the mAb 7E7 was located in the region of aa 332–531 of ASFV pB602L protein. After the second round of screening and identification, the truncated protein aa 473–531 fused to GST-Tag could be recognized by mAb 7E7 (Fig. 6B). However, upon further analysis, each of the aa 473–487, aa 473–498, aa 473–508, and aa 473–518 could be recognized by mAb 7E7, indicating that the region recognized by mAb 7E7 is located at aa 473–487 (Fig. 6C). To determine the precise epitope in pB602L recognized by mAb 7E7, we further truncated the polypeptide aa 473–487 into aa 473–482, aa 473–483, aa 473–484, aa 473–485, aa 473–486, and aa 473–487. Ultimately, these results demonstrated that one dominant antigenic epitope aa 474–482 (^474^SKENLTPDE^482^) in the C-terminus of the ASFV pB602L protein was the minimal linear epitope for mAb 7E7 binding (Fig. 6D).

### Epitope sequence analyses

**Fig. 7:**
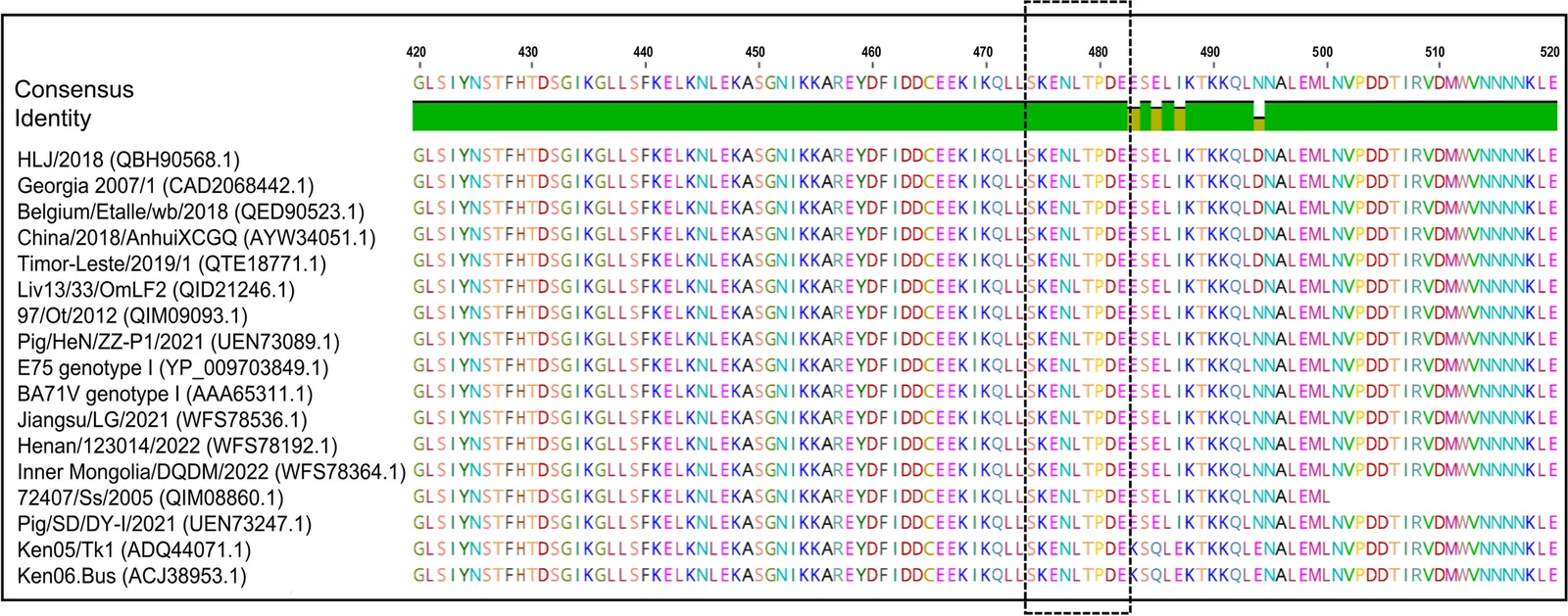
Predicting spatial structures and antigenic characteristics in the mAb 7E7 antigenic epitope. **A** Conservative analyses of novel epitopes in multiple ASFV pB602L sequence alignments. We obtained pB602L protein sequences from 17 different ASFV strains belonging to six genotypes (I, II, I and II recombinant, VIII, X, and XI genotypes) from the National Center for Biotechnology Information database and compared sequences using Geneious software. Epitope sequences are highlighted (black dotted box)

Sequence alignments were performed to explore the ^474^SKENLTPDE^482^ epitope conservation of ASFV mAb 7E7 across 17 ASFV strains (including I, II, I and II recombinant, VIII, X, and XI genotype isolates) from GenBank. As indicated in Fig. 7, ^474^SKENLTPDE^482^ was highly conserved in the tested ASFV strains and did not contain any mutation sites.

### Molecular docking analyses

**Fig. 8:**
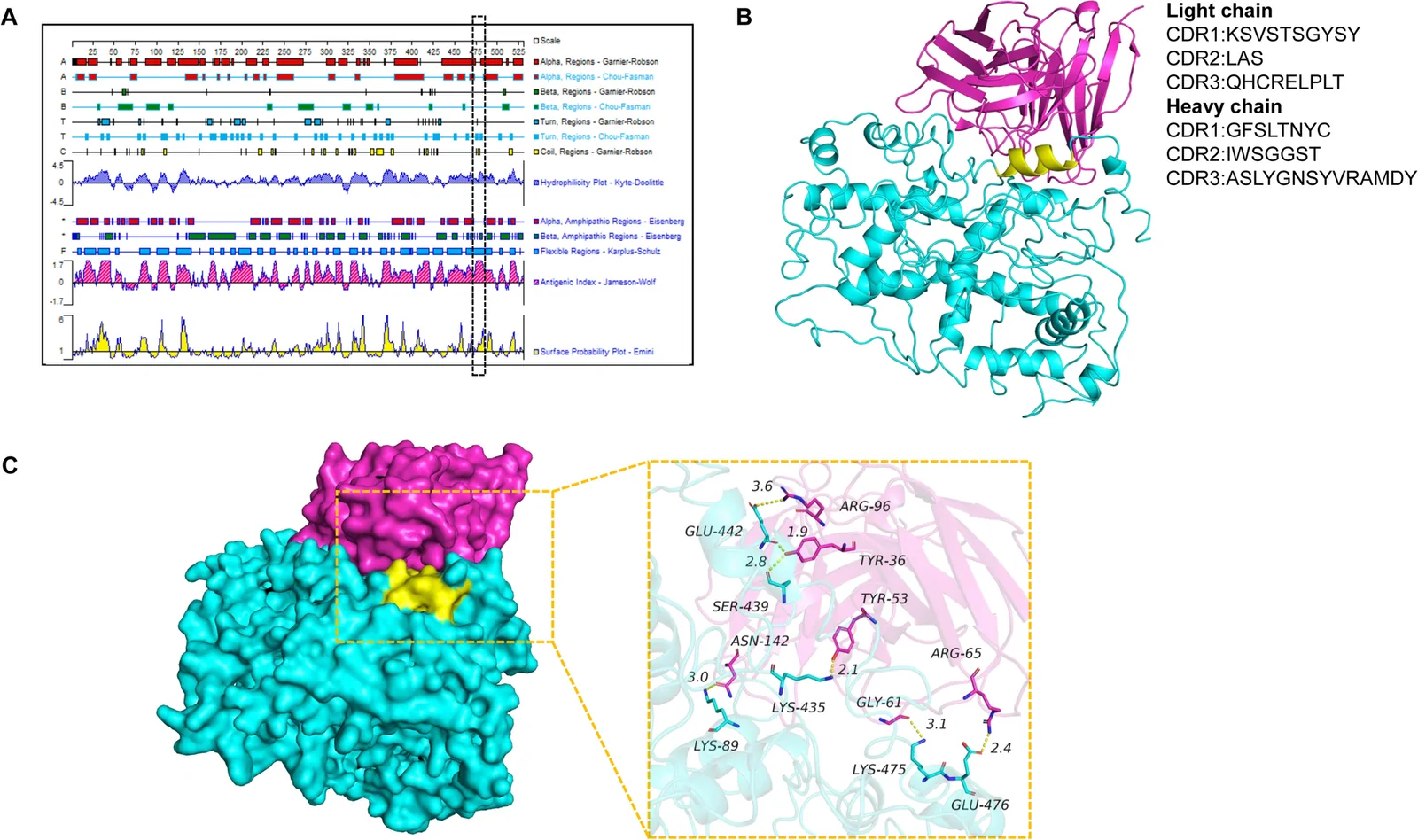
Prediction of antigenic characteristics and spatial structures of mAb 7E7 antigenic epitope. **A** Antigenic characterization of the mAb 7E7 antigenic epitope, secondary structures, and biological antigenic epitope characteristics were generated using the PROTEAN module in DNAStar. The ^474^SKENLTPDE^482^ epitope is shown (dotted box). **B** The binding mode of the pB602L complex with mAb 7E7. Predicted three-dimensional structure of the relative localization of the mAb 7E7 epitope in pB602L. Epitope amino acid residues are yellow, ASFV pB602L is cyan, and mAb 7E7 is red. The complementary determining region (CDR) of mAb 7E7 was defined using IMGT/V-QUEST. **C** ASFV pB602L and mAb 7E7 complex binding patterns are shown in the surface pattern. ASFV pB602L protein (backbone is cyan), mAb 7E7 (backbone is red). Identified regions are colored in yellow. Yellow lines represent hydrogen bonds. The structure shown in the figure was rendered using PyMOL (The PyMOL Molecular Graphics System, version 1.5.0.4, Schrödinger, LCC., New York, New York)

According to the protean prediction tool of the PROTEAN software, the 7E7 antigenic epitope of the mAb had a high hydrophilicity and a high antigenic index and contained an α-helix (Fig. 8A). The projected image shows that the 474SKENLTPDE482 epitope was exposed in 3D structures on the ASFV pB602L surface, forming a curved linear structure, shown in yellow. In addition, the mAb 7E7 clone was sequenced and the diversity of genotypes was analyzed using the IMGT/V-QUEST program (www.imgt.org/IMGT_vquest/vquest ). The results showed that the heavy and light chains were derived from IGHV1-15*01 and IGKV2D-12*01, respectively, and further analyzed the V (D) J region of mAb 7E7 and deduced the corresponding recombinant amino acid sequence of the V (D) J region, which consists of 14 and 9 amino acids for the CDR3 loop of HC and LC, respectively (Fig. 8B). To gain further insight into the interaction of ASFV pB602L with mAb 7E7, a complex docking model was generated and interaction patterns between the complexes were predicted in the molecular dynamics (MD) program MOE 2019.01. All of these interfaces are composed of a large number of non-polar and polar amino acids involved in hydrophobic and polar interactions, including salt bridges, hydrogen bonding, and hydrophobic interactions, stabilizing the dimeric interface.

## Discussion

ASFV is a highly contagious virus that has caused serious damage to swine health and the swine industry. Since its first report in 1921, no safe and effective ASFV vaccines or drugs have been developed to prevent and treat the disease (Eustace-Montgomery 1921), and scientists do not have a clear understanding of the major ASFV immunogens. Therefore, research on ASFV antigen targets could promote the development of ASFV vaccines and diagnostic reagents, as well as other prevention and control technologies. Therefore, more in-depth research on the ASFV protein structures and antigen characteristics is needed. As a highly immunogenic viral protein, pB602L is a suitable candidate for the development of diagnostic reagents. Therefore, mAbs specific to ASFV pB602L and the identification of novel linear B-cell epitopes could provide insight into ASFV pB602L antigen structures and specific anti-ASFV antibody responses. Critically, such information could be used for rational ASFV vaccine design.

The most commonly used diagnostic tests for ASFV are serologic tests. Previous studies have identified p72, p30, p54, CD2v proteins, and the polyprotein pp62 as the most antigenic proteins that induce antibodies after natural infection (Burmakina et al. 2019; Cao et al. 2021; Ren et al. 2022; Simón-Mateo et al. 1997; Zhou et al. 2023). These proteins can be used for serologic testing and vaccine development. As the major ASFV envelope protein, p72 is one of the first viral proteins to induce the antibody production after viral infection and is an important marker in ASFV diagnosis and vaccine studies (Nan Wang et al. 2019; Heimerman et al. 2018). p30 is abundantly expressed in early cell stages, has good antigenicity, and is also an important target for early virus diagnosis (Wang et al. 2022). Anti-p54 antibodies appear as early as 10 days after ASFV infection, but p54 is prone to false negative results due to amino acid sequence variations, and is not typically used as an ASF detection antigen (Alonso et al. 2001; Gao et al. 2022). The CD2v protein, a host immunoregulatory protein, is a serotype-specific cross-protective antigen of ASF and an important protective antigen against ASF (Gladue et al. 2020; Petrovan et al. 2022; Sanna et al. 2017; Song et al. 2023). pB602L is an ASFV antigen protein and its antigenic epitopes are of great importance for the development of diagnostic reagents and vaccines against ASFV. As a companion to p72 (main capsid protein), the absence of pB602L can severely alter virus assembly (Liu et al. 2019) and inhibit the processing of pp220 and pp62 polymeric proteins. In addition, pB602L is also recognized by the super immune serum of domestic and jungle pigs (Cobbold et al. 2001; Epifano et al. 2006; Gutiérrez-Castañeda et al. 2008; Reis et al. 2007). The central variable region (CVR) of the *B602L* gene shows changes in tandem repeat sequences that provide information about relationships between isolates and can be used to distinguish them (Atuhaire et al. 2013; Gallardo et al. 2014). The antigenic epitope is part of an antigen that is recognized and bound by an antibody, and the epitope-based approaches are widely used in the development of vaccines, disease diagnostics, and reagents (Evans et al. 2014; Gong et al. 2023; Khan et al. 2022; Xu et al. 2018).

ASFV pB602L is one of the most immunodominant antigens, thus, so we measured pB602L-specific IgG titers in ASFV-positive sera and showed a specific IgG titer of 1:25,600 after natural infection. mAbs are not only effective tools to combat ASFV infection, but are also powerful reagents for mapping ASFV epitopes in structural and functional studies. Using IFA and WB, mAb 7E7 showed specific responses to the inactivated ASFV Pig/HLJ/2018 strain. In addition, we observed that the novel B-cell epitope ^474^SKENLTPDE^482^ sequence, with part of an α-helix turn and coil regions, showed a strong antigen index. The binding mode of ASFV pB602L and mAb 7E7 showed that ^474^SKENLTPDE^482^ was located on the C-terminal ASFV pB602L surface, with residue interactions mainly including salt bridges, hydrogen bonds, and hydrophobic interactions, to form a stable structure. Based on its high ASFV specificity and immunogenicity, this B-cell linear epitope (mAb 7E7) can be used to develop epitope-based serological diagnostic assays.

A limitation of the current study is that live virus neutralization assays must be performed in a BSL-3 laboratory; therefore, we did not evaluate the neutralizing activity of mAb 7E7 and only verified its specificity with inactivated ASFV Pig/HLJ/2018 strain-infected PAM cells by WB assay.

In conclusion, we successfully generated a mAb (7E7) targeting the ASFV pB602L protein, which showed good specificity and sensitivity to the ASFV Pig/HLJ/2018 strain. A new linear B-cell epitope ^474^SKENLTPDE^482^ was identified at the C-terminus of ASFV pB602L, which was the minimal linear epitope for the mAb 7E7. Our study provides new biological material for ASFV studies and new methods for ASFV diagnostic development and clarifies antibody-antigen interactions.

## Data Availability

The data supporting these findings are available from the corresponding author upon reasonable request.
